# Evaluation of surface charge shift of collagen fibrils exposed to glutaraldehyde

**DOI:** 10.1038/s41598-018-28293-1

**Published:** 2018-07-04

**Authors:** Patrick Mesquida, Dominik Kohl, Orestis G. Andriotis, Philipp J. Thurner, Melinda Duer, Sneha Bansode, Georg Schitter

**Affiliations:** 1 Automation and Control Institute (ACIN), TU Wien, Gusshausstrasse 27–29, A-1040 Vienna, Austria; 2 Department of Physics, King’s College London, Strand, London, WC2R 2LS United Kingdom; 3 Institute of Lightweight Design and Structural Biomechanics, TU Wien, Getreidemarkt 9, A-1060 Vienna, Austria; 4 Department of Chemistry, University of Cambridge, Lensfield Road, Cambridge, CB2 1EW United Kingdom

## Abstract

Collagen fibrils are a major component of the extracellular matrix. They form nanometer-scale “cables” acting as a scaffold for cells in animal tissues and are widely used in tissue-engineering. Besides controlling their structure and mechanical properties, it is crucial to have information of their surface charge, as this affects how cells attach to the scaffold. Here, we employed Kelvin-probe Force Microscopy to determine the electrostatic surface potential at the single-fibril level and investigated how glutaraldehyde, a well-established protein cross-linking agent, shifts the surface charge to more negative values without disrupting the fibrils themselves. This shift can be interpreted as the result of the reaction between the carbonyl groups of glutaraldehyde and the amine groups of collagen. It reduces the overall density of positively charged amine groups on the collagen fibril surface and, ultimately, results in the observed negative shift of the surface potential measured. Reactions between carbonyl-containing compounds and proteins are considered the first step in glycation, the non-enzymatic reaction between sugars and proteins. It is conceivable that similar charge shifts happen *in vivo* caused by sugars, which could have serious implications on age-related diseases such as diabetes and which has been hypothesised for many years.

## Introduction

It has long been speculated that glycation, the non-enzymatic and uncontrolled reaction between a reducing sugar and a protein, alters the surface charge of fibrillar protein components of the extracellular matrix (ECM)^1^. This could seriously interfere with cell adhesion^2^. However, the charge alterations on individual collagen fibrils are extremely difficult to investigate experimentally as fibrils do not lend themselves easily to conventional approaches to determine electrical properties such as zeta-potential measurements in suspension.

During ageing of the organism, collagen fibrils are subject to non-enzymatic reactions that primarily affect positively charged Lys and Arg sidechains and could, thus, affect the collagen fibril surface charge. Adhesion of cells to the collagen scaffold depends on metal ion-mediated molecular interactions between transmembrane molecules and the surface of individual collagen fibrils. It is thus crucial to understand how non-enzymatic chemistry affects fibril surface charge.

*In vivo*, reducing sugars are the most common source of reactive aldehydes. Their chemistry is highly complex because, after the initial reaction step, the resulting Schiff base is highly susceptible to auto-oxidation reactions, which initiate a cascade of reactions resulting in a wide distribution of products. Furthermore, the reaction rates of physiological sugars are very low and a significant accumulation of Advanced Glycation Endproducts (AGE)^2^ occurs over the lifetime of the organism, which makes *in vitro* lab experiments difficult.

From a basic, chemical point-of-view, however, it is not only sugars that are involved in such reactions but essentially any substance that contains free carbonyl groups such as glutaraldehyde (GA), which contains two. GA is a well-known, highly reactive protein cross-linker^3^. The chemistry of GA with protein terminal amine groups is well-characterized^4^. It begins with the normal glycation reaction between its carbonyl groups and the ε-amino groups of lysine in proteins^2,3,5^ and proceeds to negatively-charged Lys-Lys crosslinks (via formation of carboxylate groups). The same, basic reaction is a hallmark of glycation^1^. We can, therefore, use GA experimentally as a model for glycation which is more reactive than typical, physiological sugars such as glucose.

Many GA-protein reaction pathways are conceivable, and it has been determined experimentally that the overall effect is a reduction of free amine content^3^. As Lys amine groups are normally present in the protonated form (-NH_3_^+^), i.e., positively charged at physiological pH, we hypothesised that free amine content reduction through Lys-Lys crosslink formation would result in a shift of the overall, net surface charge towards a more negative value.

Here, we tested this idea by exposing type-1-collagen fibrils to GA and investigating the fibril charge by Kelvin-probe Force Microscopy (KFM). KFM is a variant of Atomic Force Microscopy (AFM), which is used to map the electrostatic potential of a surface with nanometer resolution^6^. To date, KFM has been applied to investigate the charge of various, biological materials and processes. For example, it was employed to detect DNA-hybridization or avidin-biotin interactions at great sensitivity^7^, single-molecule biomolecular recognition events^8^, lipid film imaging^9^, or to map the surface charge distribution of amyloid fibrils^10^. In regards to collagen, while there is ample literature reporting AFM imaging and nanomechanical measurements^11–21^, there is very little literature on their surface charge^22^, and the influence of chemical treatments on the charge has never been investigated at the single-fibril level by KFM.

## Results and Discussion

**Figure 1:**
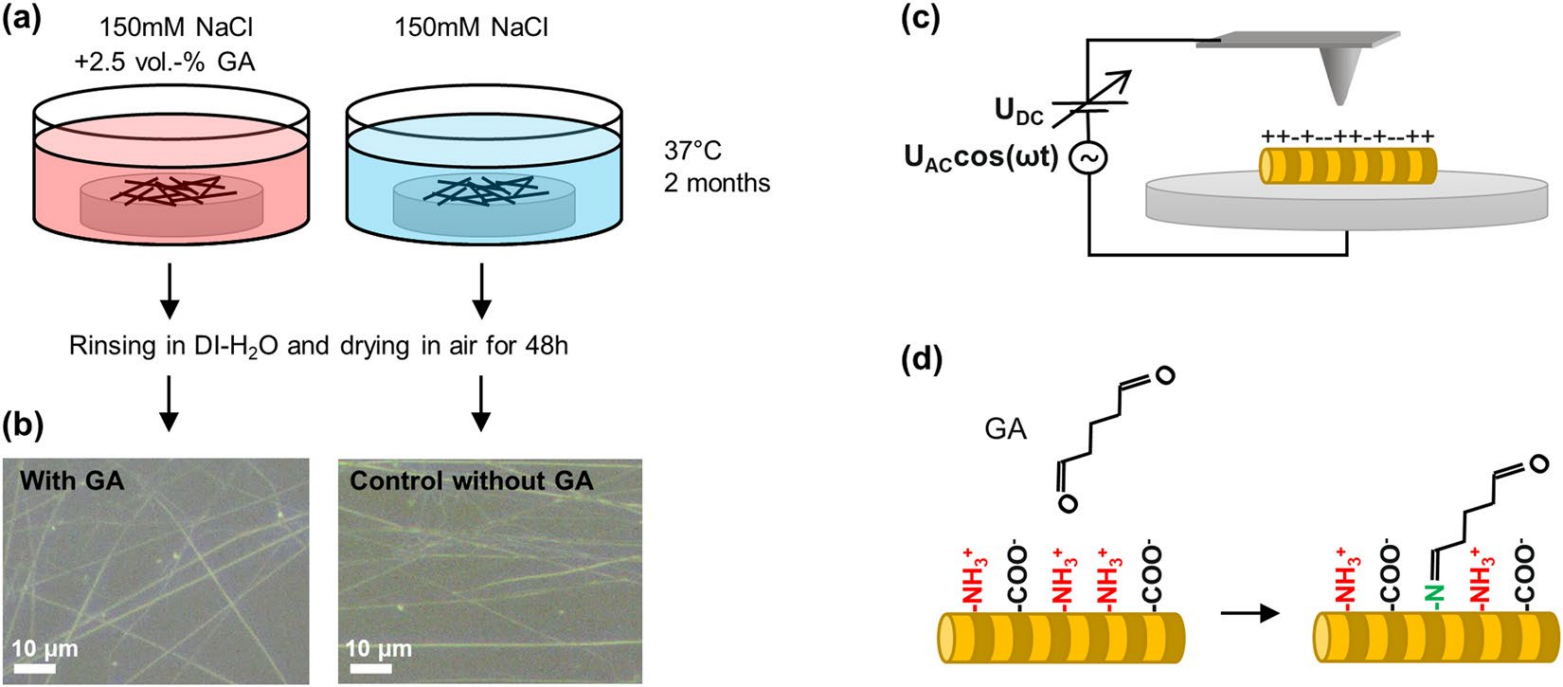
Sample preparation and experiment overview. Sample preparation (**a**), optical microscopy images of collagen fibrils (**b**), dark-field, reflected-light optical microscopy of fibrils on glass (dry samples in ambient air without cover glass, 50 × NA0.75 objective), KFM in air (**c**), and simplified, molecular mechanism of charge alteration (**d**), carbonyl groups of GA react with amine groups of collagen, with the net result of a shift of the chemical equilibrium from predominantly positively charged amine groups towards neutral species.

We used two samples of type-1-collagen fibrils from the same source: One was immersed in a GA-containing solution and one in a control solution without GA (Fig. 1a). After removing the samples from the respective solutions, we first checked them by optical microscopy (Fig. 1b): Individual fibrils (or small bundles) could readily be observed and did not show any evidence of significant degradation.

However, in KFM, we observed that the measured surface potential of a given fibril (surface potential on fibril minus surface potential of surrounding glass) changed significantly towards smaller magnitudes over the first few hours. We attribute this to drying of the sample, that is, water slowly desorbs from the fibrils over several hours. As surface water is known to affect KFM signals^23^, we left the samples to dry under ambient lab conditions for at least 48 h before conducting any conclusive KFM imaging.

**Figure 2:**
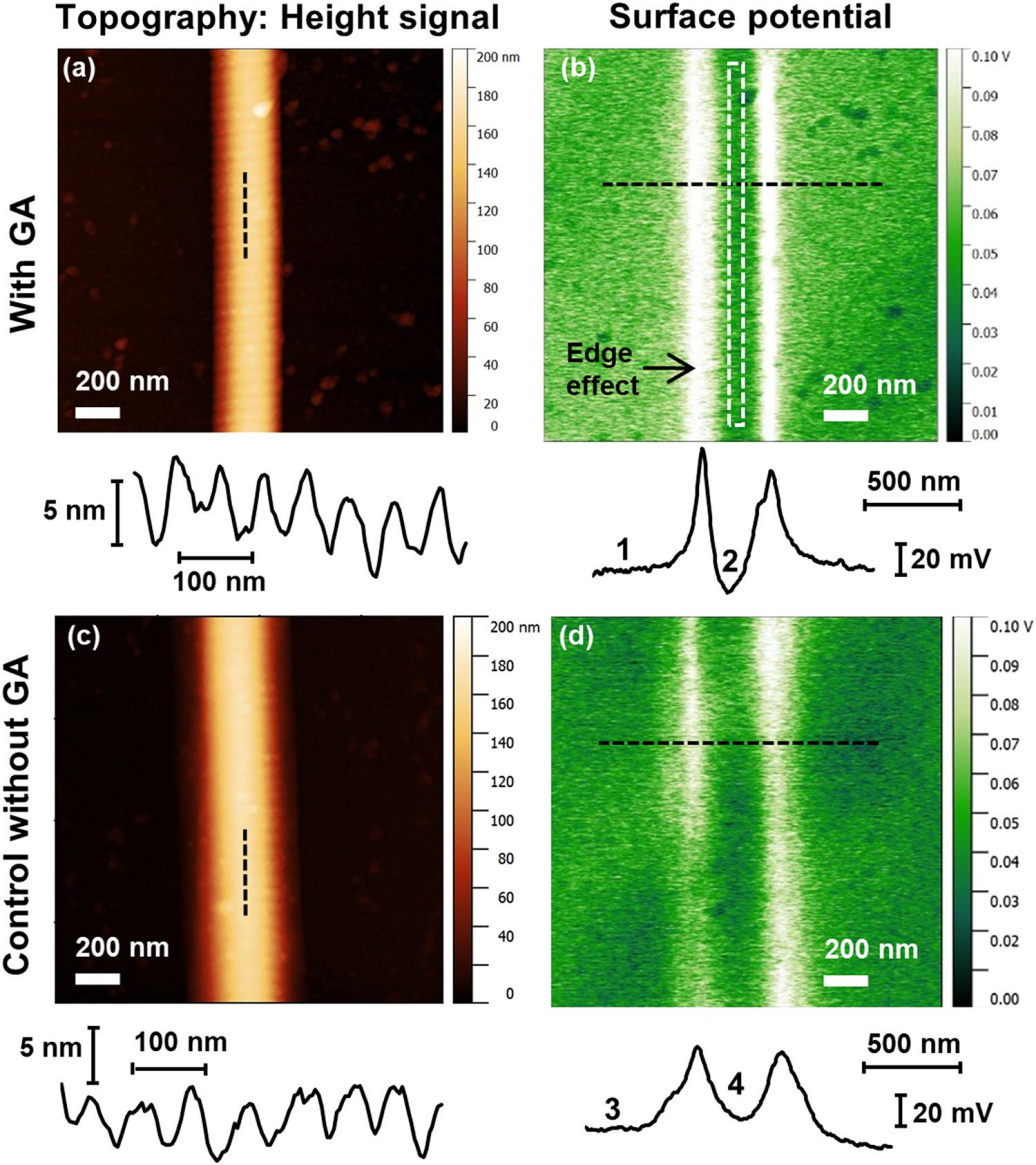
Representative AFM/KFM images of collagen fibrils. Topography (**a** and **c**) and surface potential (**b** and **d**) of randomly selected collagen fibrils on glass. Top row (**a** and **b**) sample exposed to GA-solution; bottom row (**c** and **d**) sample exposed to control solution without GA. Images taken >48 h after removal of the sample from the solutions and drying in ambient conditions (ca. 40–50% relative humidity). Below the images: Longitudinal topography profiles as indicated in the images (**a** and **c**), cross-sectional potential profiles as indicated in the images (**b** and **d**). The white, dashed rectangle in (**b**) shows an example of the approximate area which was used for determining the fibril potential in the image analysis avoiding the “edge effect”.

Figure 2 shows representative AFM topography and KFM surface potential images of a fibril on either sample. The topography images, show the usual banding-pattern of 67 nm periodicity. The height of the overlap “bumps” is unchanged upon GA exposure. This indicates that our GA treatment does not significantly affect the overall morphology of the fibrils and it is likely that the molecular structure is fundamentally preserved.

The corresponding KFM surface potential images show that there is a strong edge effect (Fig. 2b,d). That is, the left and right edge of the fibrils show peaks in the apparent surface potential. This is very common in KFM and is usually due to topography cross-talk and tip convolution when an object has dimensions similar to those of the AFM tip.

There is, however, a significant difference in the potential of the central part of the fibrils with respect to the surrounding glass. The central part of the GA-exposed fibril (2, Fig. 2b) shows a uniform potential a few tens of mV lower ( = more negative) than glass (1, Fig. 2b), whereas the central part of the control fibril (4, Fig. 2d) shows a potential a few mV higher ( = more positive) than glass (3, Fig. 2d). As the overall geometry of the two fibrils, for example their diameter as seen from the topography images, is roughly the same, and the same tip and scanning parameters (e.g. lift height) were used for all images, any tip convolution effect is the same. Thus, the difference of the surface potential along the central part of the fibrils in Fig. 2b,d is solely an effect of the GA treatment.

**Figure 3:**
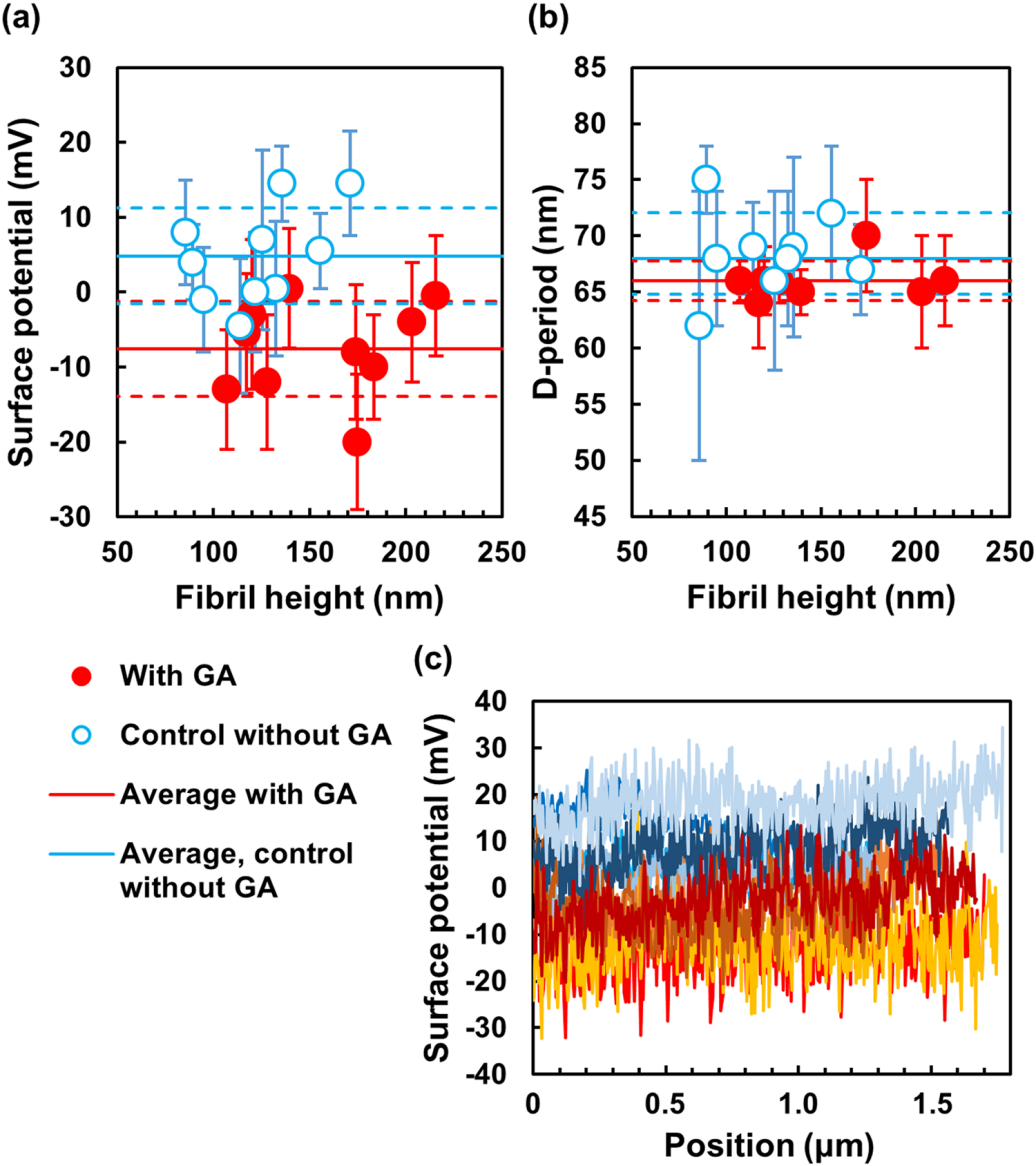
Effect of GA on surface potential and D-banding pattern of collagen fibrils. (**a**) Potential vs height of N = 10 randomly selected, individual fibrils for each sample, each data point represents one fibril with the potential determined over a length of at least 1.5 μm; (**b**) D-period vs height of the same fibrils as in (**a**), D-period determined as average over 10 bands per fibril, some fibrils had to be omitted as the banding-pattern was not pronounced enough in the AFM height images; red, full circles = sample exposed to GA; blue, open circles = control sample without GA; the horizontal error bars for the fibril heights are not shown as they are smaller (<10 nm) than the data symbols here;. Continuous, horizontal lines = average potential (**a**) and average D-period (**b**) of the data points for each sample; dashed, horizontal lines = upper and lower limit of the standard deviation of the data points. (**c**) Longitudinal potential profiles of 5 randomly chosen fibrils of each sample; profiles in shades of red/orange represent GA-exposed fibrils; profiles in shades of blue represent fibrils from the control sample. Note: The zero-potential is arbitrary; the vertical axes represent fibril potential minus glass potential.

Figure 3a shows a quantitative and statistical analysis of the effect of GA on the surface potential. Measurements with N = 10 individual fibrils on each sample show that the average surface potential of collagen fibrils is reduced, that is, shifted towards a more negative value for GA-exposed fibrils. This reduction amounts to 12.4 mV ± 6.3 mV (p = 0.0004, unpaired, two-tailed t-test of GA vs control data).

The average fibril height is 156 nm ± 39 nm for GA-exposed sample and 123 nm ± 28 nm for the control sample (Fig. 3a). This indicates no obvious effect of GA on the fibril height. The height is representative of the fibril diameter if we assume that fibrils possess a circular cross-section. The large scatter of the data points simply shows the variation of height between individual fibrils.

Figure 3c shows longitudinal profiles of the surface potential of five randomly selected fibrils of each sample. The profiles illustrate that there is some variation between different fibrils in a sample but only little variation of the potential on an individual fibril over axial distances of at least 1.5 μm, which corresponds to more than 20 banding-periods. Overall, the profiles show that the fibrils exposed to GA (red/orange profiles) exhibit a significantly more negative potential than the fibrils exposed to the control solution without GA (blue profiles).

It should be noted that the experiments in this work were performed in air (at ambient conditions). This is because KFM in the classical implementation (Fig. 1c) cannot be applied in water due to the inevitable bias voltage that needs to be applied to the tip^24^. In water, this would lead to unwanted, electrochemical reactions^25^. However, for surfaces that expose fixed charges on chemical surface groups (such as NH_3_^+^ in biological systems), the surface potential measured by KFM in air is representative in sign and magnitude of the surface charge in water^22^. In this case, the KFM potential determined in air is essentially dependent on the immediate “history” of the sample.

In order to check for any significant alteration of the fibril structure, we also analysed the D-banding period for each fibril investigated (Fig. 3). No statistically significant effect of GA exposure on the D-period was observed (p = 0.11, unpaired, two-tailed t-test of GA vs control data).

As the only difference between the GA-exposed and the control sample is the presence or absence of GA (and its reaction products), respectively, we can attribute the reduction of the surface potential to a reduction of the number of ionised amine groups. Moreover, it is also conceivable that reaction products that contain aldehyde groups can undergo hydrolysis to form carboxy groups^3^, which are normally negatively charged at neutral pH. Hence, the formation of such carboxy groups could also add to the overall shift of the surface potential to more negative values.

These results are the first, direct physical evidence that the charge of collagen fibrils is altered upon upon exposure to carbonyl-containing compounds. Such an alteration of the surface charge of collagen fibrils (and, possibly, other fibrillar proteins such as elastic fibers, if subjected to similar chemistry) is very likely to affect a range of very fundamental, physiological processes and phenomena, most importantly cell adhesion, which occurs via metal-ion-mediated interactions between transmembrane integrins and collagen fibrils. For example, reduced interaction of collagen with endothelial cells could lead to severe, vascular dysfunction. As another example, platelet cell interaction with collagen in damaged tissue is crucial for proper wound-healing and could be affected by surface charge alterations in the ECM^2^. Interference with cell adhesion is readily understandable on the molecular scale: Lys and Arg are the main residues affected by glycation and, especially, Arg is crucial as it is part of the well-known Arg-Gly-Asp (RGD) cell recognition motif for integrin receptors^2^.

## Conclusion

In conclusion, we showed, using KFM as a direct, physical method, that the net charge of collagen fibrils is shifted towards a more negative value upon exposure to a carbonyl-containing compound. In view of the wide-ranging implications that this could have, it is crucial to investigate further the effect of more physiological sugars (glucose, ribose, etc) on the electrostatic properties of ECM components on the fibrillar and molecular scale, not only in order to understand disease mechanisms but also to support the development of new treatments and therapeutic interventions.

## Methods

### Sample preparation

The source of type-I-collagen fibrils was tendon from the tail of a 6-month old, female wild-type mouse. The tail was recycled waste material, which would have otherwise been discarded and which was obtained from another institution. All local laws and regulations pertinent to research with vertebrate tissue were observed and approval was not required for this study. Bundles of collagen fibrils were manually extracted from the tendon (which was previously stored at −80 °C for several months) using tweezers. The bundles were then manually drawn over a microscopy glass cover disc (12 mm diameter; <0.2 mm thickness), which was previously cleaned by rinsing in ethanol and glued to a steel disc with silver paint to ensure good electrical contact. By performing a circular motion and dragging the bundle over the glass, many individual collagen fibrils were “left behind” on the glass surface due to the high stickiness of collagen. This procedure essentially untangles the bundles into individual fibrils or small bundles of 2–3 fibrils.

Two samples were produced with tendon from the same animal: The first sample was immersed in an aqueous solution containing 2.5 Vol.-% GA (Electron Microscopy Sciences, Hatfield PA, USA) and 150 mM NaCl, whereas the second sample was immersed in a 150 mM NaCl solution without GA and, therefore, acted as control sample. Both samples were then kept at 37 °C for 2 months in their respective solutions.

### AFM/KFM

The samples were removed from their respective solutions, thoroughly rinsed with deionised water and dried by blowing a stream of ambient air over them for a few seconds. AFM/KFM was performed with a Dimension FastScan AFM (Bruker Corporation, Billerica MA, USA) in ambient air (relative humidity = 45%, temperature = 23 °C). The same individual AFM tip (Bruker TAP150A, n-doped (Sb) Si, nominal cantilever spring constant = 5 N/m, nominal tip radius = 10 nm) was used for all AFM/KFM measurements.

Topography-mapping was performed in Tapping-Mode. Amplitude-Modulated KFM (AM-KFM or AM-KPFM) was performed at a lift height of 30 nm using an external control system for the KFM signal. This system consisted of an off-the-shelf function generator, which applied a sinusoidal signal with the cantilever’s first resonance frequency to the tip via the *tip bias* input of the Bruker Signal Access Module (SAM). The resulting deflection signal of the tip oscillation was taken from the SAM and fed into an external lock-in amplifier (7270 DSP, Ametek Inc, Berwyn PA, USA) to determine the inphase amplitude component of the oscillation (in phase with the signal from the function generator). This signal is then the error signal of the control circuit. A custom-made analogue-controller was used to perform the standard AM-KFM control procedure^26^ and the control signal, which represents the actual surface potential to be determined, was fed back to the tip via the SAM. The control signal was also input to the customisable *Input1 port* of the AFM, where it was digitised by the Nanoscope V controller and the data acquisition system (Bruker Nanoscope) for image analysis. Tuning was performed and the correct function of the KFM set-up was checked using the procedure described in the literature^27^.

Ten individual fibrils per sample were randomly selected from the live, optical video camera image of the AFM. After positioning the tip and setting the scan-direction such that the fibrils showed in vertical direction in the AFM images, all images were taken at a tip velocity of 2 μm/s with 512 pixels/line ( = ca. 4 nm per pixel).

### Data analysis

All image data was analysed using the free, third-party data analysis software Gwyddion (gwyddion.net). Topography (height signal) as well as potential images were 1st-order line-levelled using the *align rows/median* function and excluding the fibril itself from the calculation of the levelling-lines by using the *mask* function.

The fibril height was determined by taking a cross-section profile perpendicular to the fibril axis, and averaging 128 such profiles directly next to each other using the *extract profiles* function. The D-banding period was determined by taking a cross-section profile along the fibril axis and manually measuring the lengths of 10 adjacent periods and, then, calculating an average period value for each fibril.

The fibril potential was determined by selecting a rectangle not more than 10 pixels wide and along the entire length and in the exact centre of the fibril in the image (white, dashed rectangle in Fig. 2b). In this rectangle of approximately 10 × 500 pixels (equivalent to ca. 40 nm × 2000 nm), the average potential value was calculated using the *statistical quantities* function. The same was performed to the left and to the right of the fibril on the glass background in the image and away from the edge effect. The potential value of the glass (average of left and right values) was then subtracted from the potential value of fibril to determine the actual, net fibril potential shown in Fig. 3a and used for all data points.

### Data Access Statement

All data generated by this research is provided in full in the present paper.
